# Suspension Palatoplasty for Obstructive Sleep Apnea- A Preliminary Study

**DOI:** 10.1038/s41598-018-22710-1

**Published:** 2018-03-09

**Authors:** Hsueh-Yu Li, Li-Ang Lee, Eric J. Kezirian, Meiho Nakayama

**Affiliations:** 1 Department of Otorhinolaryngology - Head and Neck Surgery, Sleep Center, Linkou-Chang Gung Memorial Hospital, Taoyuan City, 33305 Taiwan R.O.C.; 2 Faculty of Medicine, College of Medicine, Chang Gung University, Taoyuan City, 33302 Taiwan R.O.C.; 3 USC Caruso Department of Otolaryngology—Head & Neck Surgery, Keck School of Medicine of USC, Los Angeles, California, 90033 USA; 4 Department of Otolaryngology, Good Sleep Center, Nagoya City University, Nagoya, 464-0083 Japan

## Abstract

Suspension palatoplasty, a new surgical technique to treat obstructive sleep apnea (OSA), has been developed to correct the retropalatal obstruction in patients with small tonsils (grade I/II) and anterior-posterior palatal (A-P) obstruction. The objecteive of this preliminary study was to investigate the effectiveness and change in retropalatal airway dimensions after suspension palatoplasty. This retrospective case series study included 25 consecutive male adults with OSA. Unique technical features of suspension palatoplasty are exposure of pterygomandibular raphe and suspension of palatopharyngeus muscle to the raphe. Six months after suspension palatoplasty, apnea-hyponea index significantly reduced from 39.8 to 15.1 (effect size = 1.6). None experienced postoperative bleeding and velopharyngeal insufficiency 1 month following surgery. Subjective **s**noring severity (visual analogue scale) and daytime sleepiness (the Epworth Sleepiness Scale) significantly improved (8.7 vs 2.0 and 10.2 vs 4.9, respectively). A-P dimension of the retropalatal airspace widened significantly on perioperative endoscopy (23.0 units vs 184.6 unites) as well as posterior air space in cephalometry (7.6 mm vs 10.2 mm). Our preliminary findings show that suspension palatoplasty seems to be an effective OSA surgery in the specific patient population with minimal complications, however, further studies including a large number of patients are required to confirm the findings.

## Introduction

Obstructive sleep apnea (OSA) is characterized by repeated obstruction of the upper airway during sleep, resulting in snoring, daytime sleepiness, impaired quality of life and possible major health-related complications^1,2^. Mainstream treatments for OSA are positive airway pressure, oral appliances and surgery. Among these, surgery is the only treatment modality to improve OSA without the use of device. Uvulopalatopharyngoplasty (UPPP) was the first surgical procedure specifically designed for snoring and OSA and remains the most commonly used operation in sleep surgery^3^. However, UPPP was criticized in low success rate and perioperative complications^4–6^. In recent years, lateral pharyngeal wall collapse is considered an important factor in the development of OSA^7^. Therefore, many alternative soft palate surgical techniques have been developed in an attempt to improve outcomes, with the focus on repositioning of tissue to stabilize the lateral pharyngeal wall and expand the lateral dimension of the velopharynx^8–12^. On the contrary, some patients with small tonsils have clear evidence of anterior-posterior (A-P) collapse in the retropalatal airway that are rarely discussed.

To further expansion of the velopharynx, Mantovani *et al*.^13^ performed roman blinds technique. Vicini *et al*.^14^ reported barbed reposition pharyngoplasty, which uses a knotless bidirectional re-absorbable stitches to outer mucosal suture the palatopharyngeus muscle with pterygomandibular raphe. Some other alternative soft palate procedures have numerous changes, and it is not always possible to sort out which changes might be beneficial.

In this article, we present a new technique and use the term of suspension palatoplasty, which was developed as a less-invasive repositioning procedure by pulling the palatopharyngeus muscle to the pterygomandibular raphe (anchor) for the suspension of the soft palate in OSA patients with A-P palatal collapse and small tonsils. The objective of this study is to examine its perioperative morbidity and effectiveness in opening the retropalatal airway and improving OSA.

## Results

### Study population

There were 25 male patients with OSA (mean age = 41.1 years [95% CI 37.0‒45.2 years] and body mass index [BMI] = 24.9 kg/m^2^ [95% CI 24.0‒25.8 kg/m^2^]). Sixteen patients had grade I tonsils (64%) and nine patients had grade II tonsils (36%). Fifteen patients had modified Mallampati grade II tongue position (60%) whereas 10 patients had grade III tongue position (40%).

### Recovery after suspension palatoplasty

**Figure 1:**
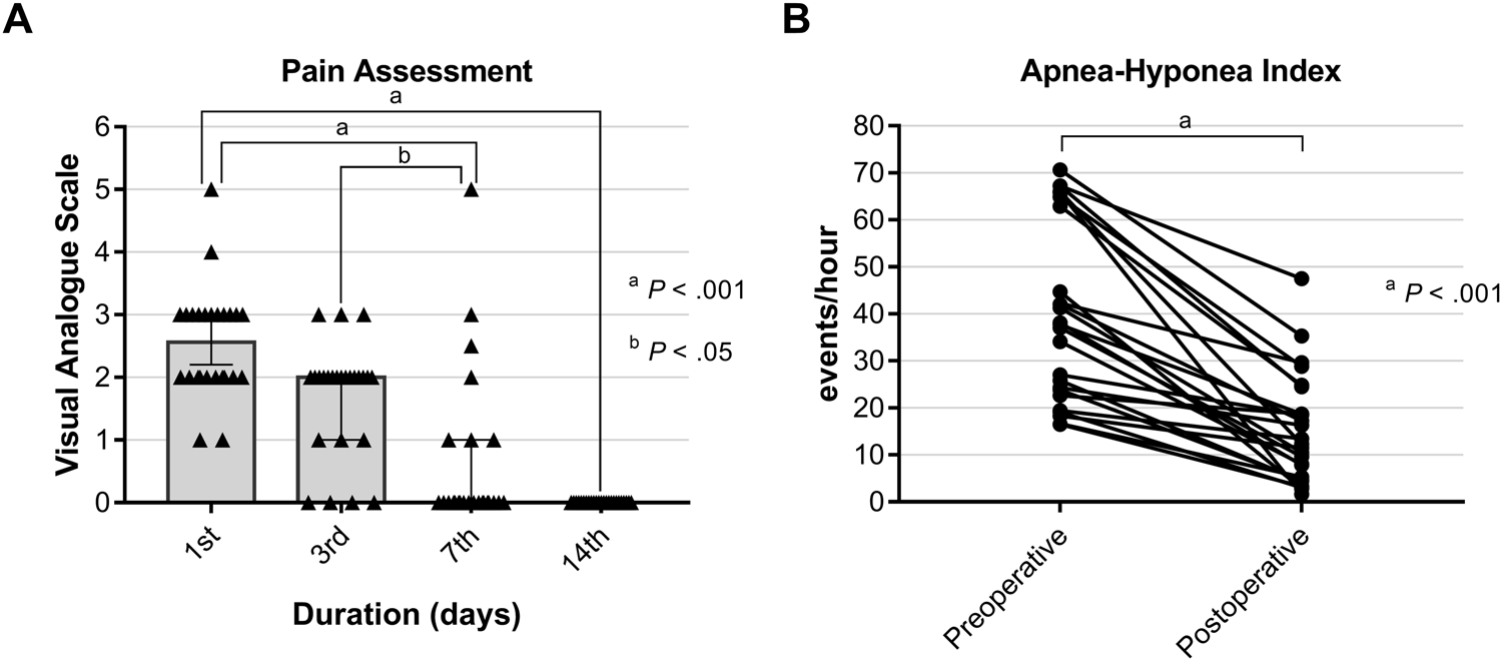
Postoperative pain profile and change of apnea-hyponea index. Postoperative pain assessment reveals that mild-to-moderate wound pain significantly improved at the 7^th^ day and completely subsided at the 14^th^ day (**A**).Comparison of apnea-hypopnea index indicated a significant reduction of the disease severity 6 months after suspension palatoplasty (**B**).

All patients were extubated at the end of the procedure and then remained hospitalized as inpatient for 3 days under the regulations of National Health Insurance in Taiwan. Postoperative pain profile revealed that mild-to-moderate wound pain significantly improved at the 7^th^ day and completely subsided at the 14^th^ day (Fig. 1A) No postoperative bleeding was noted. Additionally, there was no hypernasality, alternations in speech, or taste disturbance observed one month postoperatively. Globus sensation of the throat was common and disappeared unexceptionally after removal of retained stitches on low edge of the soft palate.

### Changes in the primary and secondary outcomes

Six months after surgery, there were substantial reductions in apnea-hypopnea index (AHI; the primary outcome measurement) (39.8 [95% CI 32.0‒47.6] vs 15.1 [95% CI 10.4‒19.9], *P* < 0.001, effect size = 1.6; Fig. 1B). The secondary outcome measurements included other sleep study parameters, as well as snoring severity and daytime sleepiness were significantly improved. There was no significant change in BMI (24.9 [95% CI 24.0‒25.8] vs 24.6 [95% CI 23.6‒25.5], *P* = 0.33, effect size = 0.27). The retropalatal airway space during perioperative 70° endoscopy (both anterior-posterior and lateral dimensions) and lateral cephalogram increased significantly (Table 1). The classical success rate was 56% (14/25), the response rate was 48% (12/25), and the cure rate was 24% (6/25).

**Table 1:** Comparison of preoperative and postoperative treatment outcomes.

Variables	Preoperative	Postoperative	Effect size
	Mean (95% CI)	Mean (95% CI)	
Subjective symptoms			
Snoring severity (visual analogue scale)	8.7 (8.3‒9.2)	2.1 (1.4‒2.9)	6.2^a^
Daytime sleepiness (Epworth sleepiness scale)	10.2 (8.4‒12.1)	4.9 (3.7‒6.1)	1.3^a^
Endoscopic measurement of the retropalatal space in perioperative period			
Anterior-posterior diameter (unit)	23.0 (15.1‒30.9)	184.6 (161.4‒207.8)	2.7^a^
Lateral diameter (unit)	167.9 (143.3‒192.5)	336.5 (314.8‒358.2)	2.6^a^
Lateral cephalometry			
Posterior airway space (mm)	7.6 (6.7‒8.5)	10.2 (9.4‒10.9)	1.4^a^
Polysmonography			
Apnea-hypopnea index (event/hour)	39.8 (32.0‒47.6)	15.1 (10.4‒19.9)	1.6^a^
Apnea-hypopnea index_supine (event/hour)	44.9 (35.3‒55.4)	17.2 (9.8‒24.6)	1.2^a^
Apnea-hypopnea index_non-supine (event/hour)	15.9 (5.6‒26.2)	3.8 (1.5‒6.1)	0.6^b^
Apnea index (event/hour)	18.5 (10.5‒26.5)	2.1 (0.8‒3.3)	0.9^a^
Minimal pulse oxygen saturation (%)	80.5 (77.2‒83.9)	87.0 (84.9‒89.1)	0.9^a^
Snoring index (event/hour)	423.6 (206.4‒640.9)	207.4 (119.1‒295.8)	0.5^b^
Sleep efficiency (%)	84.2 (79.0‒89.4)	89.1 (85.9‒92.3)	0.4^b^

Note: ^a^*P* < 0.001; ^b^*P* < 0.05; before versus after, paired Student *t* test (two-tailed).

CI, confidence interval.

## Discussion

The present study demonstrated the technique of suspension palatoplasty, which advances the soft palate by submucosal suspension of the palatopharyngeus muscle to a high and robust fascia-pterygomandibular raphe. This is the first study to report a modified palatal surgery fully dedicated to OSA patients with small tonsil and A-P palatal collapse. It’s noteworthy to comprehend that suspension palatoplasty and our previous technique-relocation pharyngoplasty were designed to treat different type of velopharyngeal obstruction. Relocation pharyngoplasty is aimed to improve sagittal collapse of the velopharynx by stabilization of the lateral pharyngeal wall. On the contrary, suspension palatoplasty is applied to ameliorate anterior-posterior collapse of the velopharynx by pulling the soft palate forward. As a matter of fact, the techniques of relocation pharygoplasty, barbed reposition pharyngoplasy and suspension palatoplasy influence and inspire each other in the evolutionary process approaching to the treatment of individual OSA patients with precise and less invasive model. The preliminary results showed minimal morbidities with significant improvement in A-P dimension of retropalatal space and OSA.

One major complication of UPPP is velopharyngeal insufficiency from the excessive excision of the soft palate^6^. Normal velopharyngeal closure needs adequate contraction of levator veli palatinus muscle and uvular muscle to separate the nasopharynx and oropharynx. Suspension palatoplasty advances the soft palate instead of cutting the palatal muscles that prevents cleft palate-like wound and consequently maintains normal pharyngeal function. In this study, no hypernasality, voice change or swallowing disturbance was reported. We presume that suspension palatoplasty not only advances but also elevates the soft palate that facilitates the contraction of levator veli palatinus muscle to close the nasopharyx during swallowing and phonation^15^. No tonsillar wound bleeding was encountered in this series. We suppose that small sample size may be the major reason. Besides, small tonsil is less likely to bleed than large tonsil (high blood supply from repeated tonsillitis) and the implementation of tension-relieving dissection reduced tonsillar wound dehiscence. The postoperative pain criticized in traditional UPPP was mild in suspension palatoplasty as shown in postoperative pain profile. The results are multifactorial and may be attributable to small tonsillar wound, limited mucosa sacrificed, no excision of the muscle, the use of low-thermal injury tool^16^, the performance of tension-releasing dissection and use of adequate pain control^17^.

The existence of globus sensation after UPPP can be attributable to “hard contact” between palatal scar and tongue during swallowing, and mucus retention due to lack of sweeping movement after excision of uvular. Suspension palatoplasty preserves the uvular muscle and palatal mucosa to eliminate the formation of globus sensation. Temporary globus sensation from retained stitches on the palatal mucosa improved dramatically after removal of the stitches.

Two major clinical manifestations of OSA including snoring and daytime sleepiness improved significantly after suspension palatoplasty. Subjective snoring severity improved significantly from 8.7 to 2.1. This result could be attributed to effective widening of the velopharynx by advancement of the soft palate as shown in nasopharyngoscopy and lateral cephalometry, increased tension of the soft palate through lateralization of posterior pillar, and decreased vibratory amplitude by shortening of the uvula. The Epworth Sleepiness Scale reduced significantly from 10.2 to 4.9 indicates noticeable improvement in patients’ daytime sleepiness. Polysomnography revealed that AHI, apnea index and minimal pulse oxygen saturation improved significantly after suspension palatoplasty. The success rate of suspension palatoplasy, although significantly increased in the retropalatal space, was only 56%. This suboptimal outcome may be attributable to primary tongue collapse that compresses the soft palate and presents as A-P palatal obstruction. In our previous study of drug-induced sleep computed tomography scan, tongue obstruction can be identified as upper (body, 30%), lower (base, 37%), and upper followed by lower (biphasic, 33%) obstruction models^18^. Upper and biphasic tongue obstruction comprise 63% in whole tongue obstruction, which are likely to be misdiagnosed as A-P palatal obstruction in endoscopic examination. Accordingly, we speculate a considerable percentage of tongue obstruction existed in this specific group patient that needs further research and treatment. Another concern is the role of BMI in surgical outcomes. The average BMI of these patients was normal or slightly overweight. Accordingly, the outcomes of suspension palatoplasty in a heavier population may decline since obesity is a poor surgical outcome predictor for OSA.

There are some differences between suspension palatoplasty and barbed reposition pharyngoplasty: (1). The pterygomandibular raphe is fully identified as the anchor and the palatopharyngeus muscle is sutured intrinsically to the raphe. (2) Suture of only upper palatopharyngeus muscle to the raphe offers adequate suspension of the soft palate without significant tension. (3) We used ordinary 2-0 Vicryl with three interrupted sutures (between raphe and palatopharyngeus muscle) to distribute the pulling strength and decrease the risk of tearing the palatopharyngeus muscle. (4) The uvular tip (non-muscular part) is removed and cauterized at the anterior aspect of distal uvular muscle to bend forward the uvula for further reduction of snoring.

There are three major limitations of this study (1). This is a retrospective study. After initial pilot cases with positive response, we established the clinical inclusion and exclusion criteria to homogenize our study population with clear treatment purpose toward a higher quality research. More importantly, this study is not a complex palate and hypopharynx procedure so as to elucidate the effectiveness of suspension palatoplasty (2). The sample size is small and without control group in study design. The small sample size prohibits subgrouping of the study population to testify the role of confounding factors in contributing to the surgical efficacy. Further, we are unable to differentiate primary palatal collapse from secondary palatal collapse due to tongue compression during endoscopic examination. A larger patient population with control for group comparison and outcome predictor analysis has been ongoing to elucidate these questions. 3. A short-term follow up. It is anticipated that surgical outcomes for OSA decline with time due to maturity of scar and relapse of the suspended airway. Accordingly, we are going to provide oropharyngeal myofunctional therapy program^19^ in our sleep surgery patients to enhance long term results.

### Summary

Using suspension technique instead of wide excision of the soft palate, suspension palatoplasty shows no major complications and minimal morbidities. The use of pterygomandibular raphe as the anchor to suspend palatopharyngeus muscle is effective to advance the soft palate and significantly enlarge the retropalatal airway in OSA patients with small tonsils. Suspension palatoplasty significantly improved subjective snoring, daytime sleepiness and OSA. However, further studies including a large sample size are required to confirm the findings.

## Methods

### Ethics statement

This retrospective study was approved by the Institutional Review Board of the Chang Gung Medical Foundation (origin number: 201700277B0) and patient informed consent was waived.

### Study population

Twenty-five male patients with OSA who underwent suspension palatoplasty in a tertiary referral sleep center at Linkou Chang Gung Memorial Hospital, Taoyuan City, Taiwan from June 22, 2016 to December 12, 2016 were retrospectively reviewed. Candidates for suspension palatoplasty were diagnosed adults with OSA (AHI > 5 events/hour on preoperative sleep study) and snoring with or without daytime sleepiness. All subjects failed conservative treatments (body weight reduction, positional therapy) and continuous positive airway pressure therapy. Inclusion criteria included: age between 20 and 50 years, BMI < 35 kg/m^2^, small tonsils (tonsil size grade I or II)^20^, coronal type and total or nearly total A-P collapse of the velopharynx according to VOTE classification during the endoscopic examination (drug-induced sleep endoscopy^21^ or Muller’s maneuver^22^), mouth opening space ≥3 finger breadth (4 cm). Exclusion criteria included significant retrognathia affecting airway, Friedman tongue position IV^20^, lingual tonsil or epiglottis obstruction during endoscopic examination, severe medical disease, previous laser-assisted uvuloplasty, unfit for general anesthesia (American Society of Anesthesiologists physical status class > 2).

### Surgical procedure

**Figure 2:**
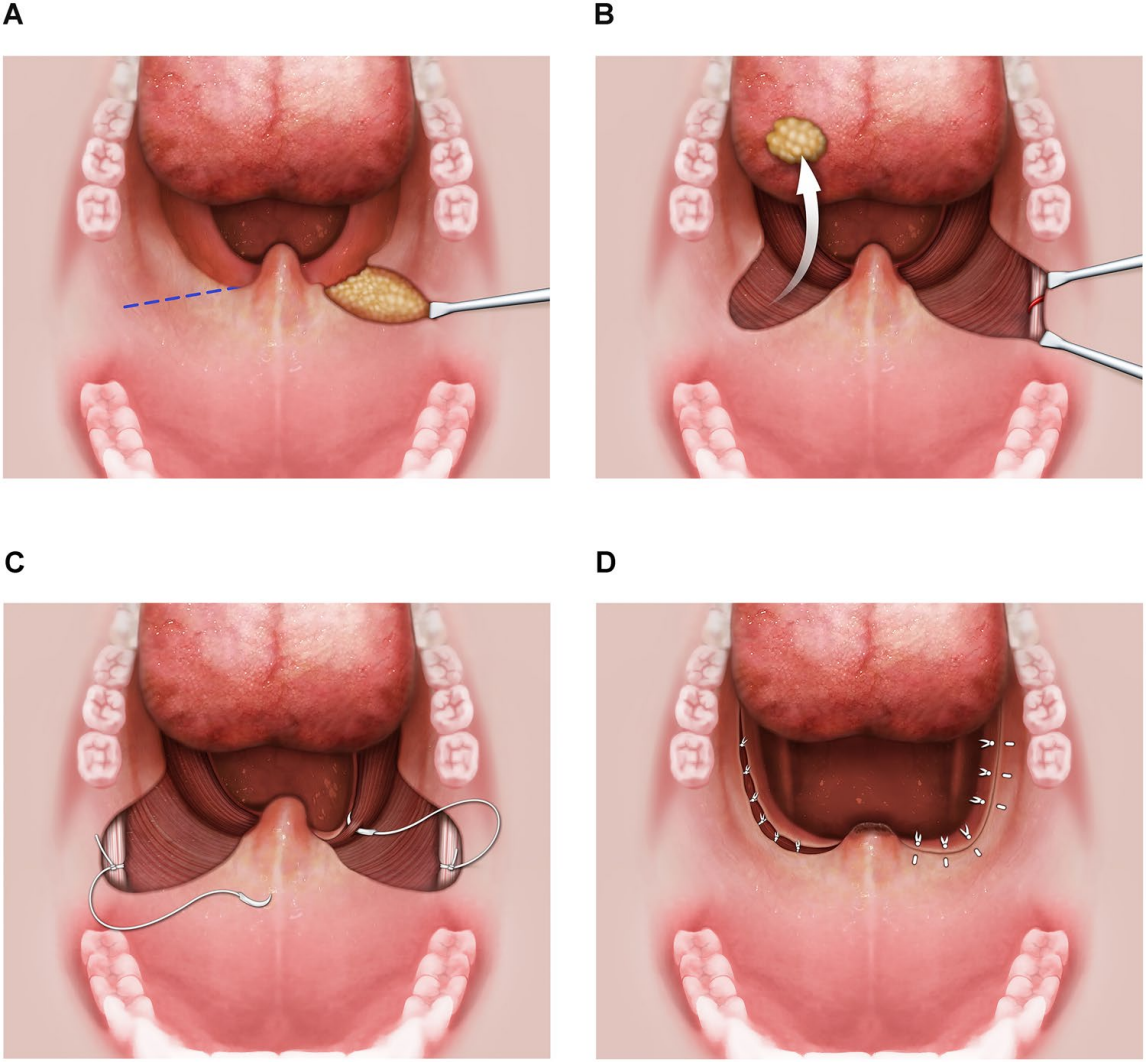
Surgical procedure of suspension palatoplasty. Linear mucosal incision, exposure of supratonsillar adipose tissue (**A**). Removal of supratonsillar fat, tonsillectomy, and exposure of pterygomandibular raphe (**B**). Suture of the raphe as anchor then suture the palatopharyngeus muscle for suspension (**C**). Repeated suspensions, closure of tonsillar fossa, mattress suture of posterior and anterior pillar, partial uvulectomy (**D**).

**Figure 3:**
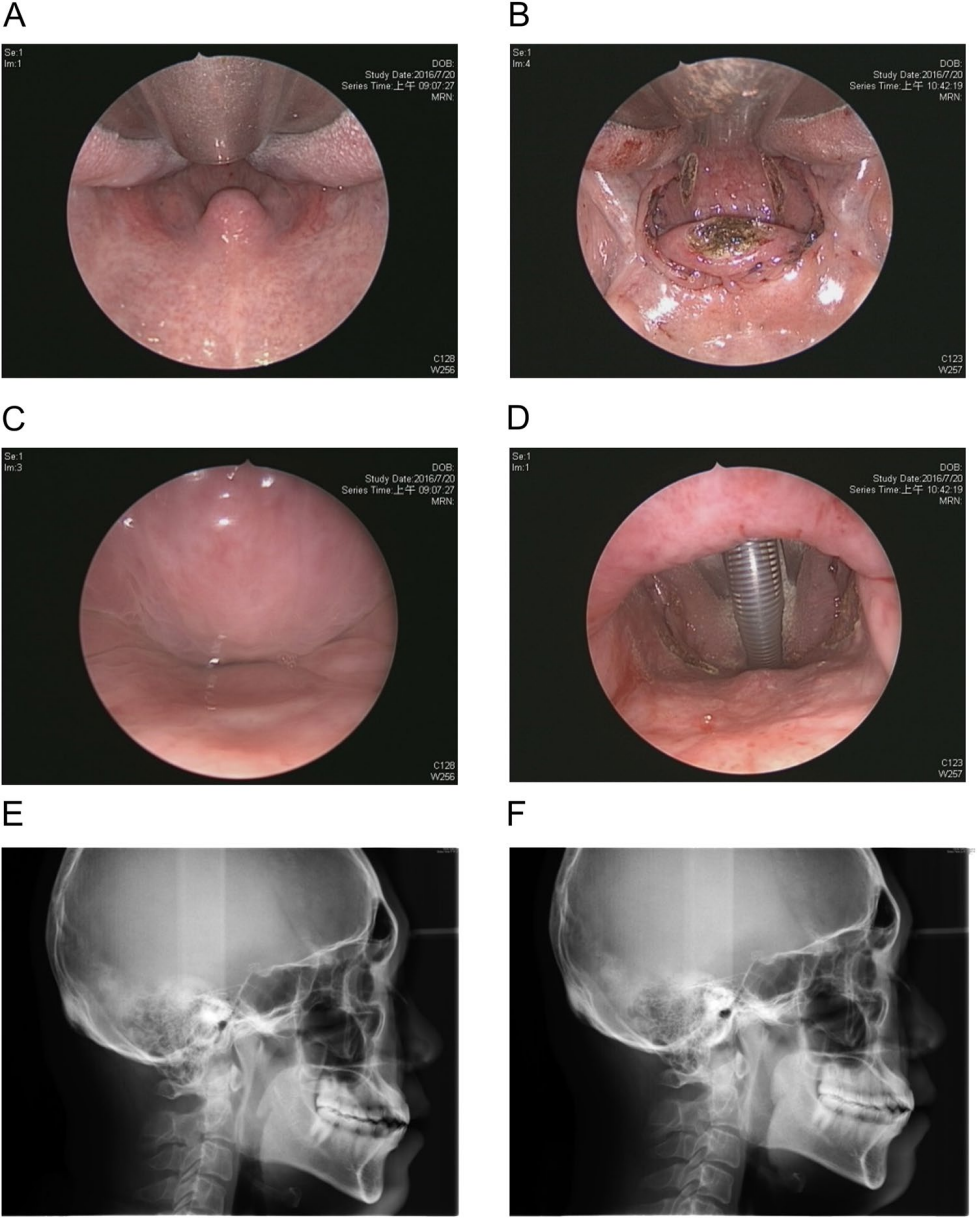
Changes of the upper airway after suspension palatoplasty. Perioperative intraoral view (**A**,**B**) and transnasal view of velopharyngeal airway (**C**,**D**) and lateral cephalometry (**E**,**F**) before (**E**) and one month after (**F**) suspension palatoplasty.

Suspension palatoplasty was performed under general anesthesia with oral endotracheal intubation. Patients had their heads extended and an adequately size of mouth gag was used to expose the oropharynx. Surgical tool used for tissue dissection and hemostasis was plasma knife (model: cutting-4, coagulation-6). The operation was initiated by a linear mucosal incision from the point of anterior pillar touching the uvula to 1 cm in front of center mark of pterygomandibular raphe. Submucosal fat tissue in this semilunar-shaped supratonsillar area was dissected from the underlying muscles and removed (Fig. 2A). Tonsillectomy was then performed with careful preservation of palatopharyngeus muscle and pillar mucosa. The supratonsillar mucosa was elevated laterally to expose the pterygomandibular raphe, a whitish firm fascia that can be easily distinguished from surrounding soft tissue (Fig. 2B). Generally, a vessel traversing the raphe was identified, and this was cauterized as a precautionary measure. Using 2-0 Vicryl, a deep layer of 3 interrupted 2-0 Vicryl sutures was placed to secure the palatopharyngeus muscle to the pterygomandibular raphe. Each suture was passed through the pterygomandibular raphe first and then through the palatopharyngeus muscle, starting superiorly in the upper one-third of the palatopharyngeus muscle, adjacent to the lateral aspect of the musculus uvulae. Subsequent sutures were then placed at a distance of 5 mm inferiorly (Fig. 2C). The lower part tonsillar fossa was then closed by suturing the palatoglossus, superior pharyngeal constrictor and palatopharyngeus muscles together. The posterior pillar is sewn onto the anterior pillar with vertical mattress sutures. The same steps are repeated on the opposite side, and, finally, the distal (non-muscular) part of the uvula is resected (Fig. 2D). Vertical cuts in the posterior pharyngeal wall mucosa and submucosal tissue are performed to release tension if there is formation of horizontal tension band after closure of bilateral tonsillar fossae. Perioperative changes of oropharyngeal structure (0° rigid endoscope, intraoral), retropalatal space (70° rigid endoscope, trans-nasal), and posterior air space (lateral cephalometry) in a patient are demonstrated in Fig. 3.

### Outcome measurement

Change of AHI six months after suspension palatoplasty was the primary outcome. Other outcomes included changes of polysomnographic indices (e.g. AHI_supine, AHI_non-supine, apnea index, minimal oxygen saturation, snoring index, and sleep efficiency); perioperative change in retropalatal space during 70° rigid endoscopy; retropalatal airway space on lateral cephalometry; daytime sleepiness as measured by the Epworth Sleepiness Scale (range, 0–24)^23,24^; snoring severity (visual analogue scale; range, 0‒10) assessed one month postoperatively, and postoperative pain (visual analogue scale; range, 0‒10). Classical success (Sher’s criteria^4^) was defined as reduction of AHI > 50% and postoperative AHI < 20^4^. Response was defined as a > 50% reduction in AHI and postoperative AHI < 15, postoperative ESS < 10, and postoperative snore-VAS < 5. Cure was defined as a > 50% reduction in AHI and postoperative AHI < 5.

### Statistical analysis

Continuous and ordinal data are presented as mean and 95% CI and were compared using the paired Student *t* test (for comparing medians between baseline and postoperative values). Categorical data are presented as numbers and percentages and were compared using the Fisher’s exact test. Two-tailed *P values* < 0.05 were considered statistically significant. All statistical analyses were performed using Graph Pad Prism 7.00 for Windows (Graph Pad Software Inc., San Diego, CA, USA) and G*Power 3.1.9.2 software (Heinrich-Heine University, Dusseldorf, Germany).
